# Cryopreservation of ram semen: baicalein efficiency on oxidative stress, chromatin integrity, viability and motility post thaw

**DOI:** 10.3389/fvets.2024.1394273

**Published:** 2024-04-05

**Authors:** Fatih Avdatek, Şükrü Güngör, Mehmet Fuat Gülhan, Muhammed Enes İnanç, Kemal Tuna Olğaç, Barış Denk, Deniz Yeni, Umut Taşdemir

**Affiliations:** ^1^Faculty of Veterinary Medicine, Department of Reproduction and Artificial Insemination, Afyon Kocatepe University, Afyonkarahisar, Türkiye; ^2^Faculty of Veterinary Medicine, Department of Reproduction and Artificial Insemination, Mehmet Akif Ersoy University, Burdur, Türkiye; ^3^Technical Sciences Vocational School, Department of Aromatic Plants, Aksaray University, Aksaray, Türkiye; ^4^Faculty of Veterinary Medicine, Department of Reproduction and Artificial Insemination, Ankara University, Ankara, Türkiye; ^5^Faculty of Veterinary Medicine, Department of Biochemistry, Afyon Kocatepe University, Afyonkarahisar, Türkiye; ^6^Faculty of Veterinary Medicine, Department of Reproduction and Artificial Insemination, Aksaray University, Aksaray, Türkiye

**Keywords:** ram semen, cryopreservation, baicalein, chromatin damage, antioxidant, oxidative stress

## Abstract

Baicalein (B) has potential antioxidant properties, but it has not been tested as a ram semen extender. This study aimed to assess the impact of B on various sperm parameters and determine its potential influence on semen quality after the freeze-thawing process. During the breeding season, ejaculates were obtained from four rams with the aid of an artificial vagina. The collected mixed semen samples were divided into four groups: control (C; 0), B0.5 (0.5 mM), B1 (1 mM), and B2 (2 mM). After semen extension, the samples were loaded into 0.25 mL straws and stored for 2 h at 4°C prior to freezing in liquid nitrogen vapor and thawed in a water bath at 37°C. Among the groups, B0.5 demonstrated the highest progressive motility results, while B1 and B2 exhibited reduced motility (*p* < 0.05). In terms of high mitochondrial membrane potential, plasma membrane and acrosome integrity, and viability, B0.5 showed significantly superior outcomes to the other B groups (*p* < 0.05), although it was not significantly better than C. B1 displayed the highest plasma membrane integrity levels (*p* < 0.05). Notably, B2 displayed the lowest total antioxidant status levels among the groups (*p* < 0.05). The findings of this study suggested that the *in vitro* spermatological characteristics of ram spermatozoa such as progressive motility and chromatin integrity can be protected from the freeze-thawing process by using the 0.5 mM dose of baicalein as a semen extender. The treatment of sperm freezing might benefit from further in-depth research on the role of B in the improvement of cryoinjury and its underlying processes.

## 1 Introduction

Semen freezing is a technique that is employed to preserve sperm cells for future utilization in assisted reproductive technologies (1, 2). However, different freezing techniques can impact sperm cell viability and motility, and proper handling and storage conditions are essential to preserving the quality of sperm cells over time (3–5). The addition of antioxidants in extenders is a potential approach to reducing cryo-damage (6, 7).

Reactive oxygen species (ROS) are chemicals that can degrade DNA, proteins, and cell membranes. Antioxidants are molecules that can counteract the effects of ROS (8). Several studies have examined the use of antioxidants, including vitamin E, vitamin C, and melatonin, in semen cryopreservation (9–11). It has been reported that adding antioxidant supplements increases post-thaw sperm cell viability, motility, and DNA integrity.

As a natural flavonoid and aglycone, Baicalein (B) can be found in the roots of the Chinese plant *Scutellaria baicalensis* (12). B has the chemical formula C_15_H_10_O_5_ and a relative molecular mass of 270.24 g/moL. Researchers have studied B for its anti-inflammatory, antioxidant, and anticancer potential (12). Additionally, it has been demonstrated that B has antimicrobial activity against a variety of pathogens, such as bacteria and viruses. The possibility of using it to treat neurological illnesses, including Alzheimer’s and Parkinson’s, has also been explored (13–15).

Furthermore, B can block the production of ROS by inhibiting xanthine oxidase activity (16). Hydrogen peroxide and other peroxides can damage neurons. B has a greater antioxidant activity and considerable protective benefits against H_2_O_2_-induced oxidative damage in human neural cells (17). A significant improvement in cell viability and mitochondrial protection through a redox-dependent mechanism has been demonstrated in studies on cellular toxicity. Authors have suggested that B plays an important role in mitochondrial functioning, it reduces apoptosis and the mitochondrial membrane potential, inhibits caspase activation, improves the production of ATP, and triggers the consumption of ADP (13, 14, 18, 19).

Several studies have investigated the impact of B on cell signaling pathways involved in mitochondrial functions in the context of maintaining mitochondrial physiology and determining the fate of cells (14, 20–22). B may have antioxidant properties that can be beneficial for reproductive health, but further research is required to fully appreciate its potential advantages in this regard. Cryopreservation of ram’s semen using B as a semen extender has not been studied. Given this information, this study aims to evaluate the effects of B on ram semen by measuring sperm motility, chromatin damage, oxidative stress, and other antioxidant parameters to reduce the likelihood of cryo-damage caused by freeze-thawing.

## 2 Materials and methods

### 2.1 Study design

The Animal Research Ethics Committee of Afyon Kocatepe University (Approval No. 49533702/333) approved the protocol of this study. Four Sönmez (25% Chios and 75% Tahirova) breed rams, aged 2–3 years, were included in the experiment. During the reproductive season, 28 ejaculate samples were collected from four males, twice a week, with the aid of an artificial vagina. The ejaculates which had >80% motility, ≥1.5 mL volume, and ≥ 1×10^9^ spermatozoa/mL were used for the study. The ejaculate samples were mixed for the procedures and analyses. Four distinct groups were created to study the effects of B (item no. 70610, 95%, Cayman Chemical Company, Michigan, United States). The groups included the experimental groups in which 0.5 mM, 1 mM, and 2 mM doses of B were added as semen extenders (B0.5, B1, and B2, respectively) and a control group (C). The mixed ejaculates that were separated into four aliquots and extended using a Tris-based extender (3.63 g Tris [T1503], 0.5 g fructose [F0127], 1.82 g citric acid [C0759]/100 mL double-distilled water) to dilute them, as well as 15% egg yolk and 6% glycerol. To produce the groups, 2 mM of B was dissolved in 1 mL of ethanol (Merck, 99%). Extended semen equilibrated at 4°C for 2 h, after equilibration held in liquid nitrogen vapor (11 cm above the liquid nitrogen, −110 to 120°C) for 12 min. Then the samples were frozen in a flow of liquid nitrogen vapor and placed in a container of liquid nitrogen for long-term storage. Following the cryopreservation process, the frozen semen samples were subjected to thawing in a water bath set at 37°C for a duration of 30 s, after which they were considered ready for analysis. Progressive motility, total motility, and kinetic spermatozoon parameters were evaluated with a computer-assisted sperm analyzer (CASA; MICROPTIC S.L., Sperm Class Analyzer software, SCA^®^ v.4.2; Spain) system. Mitochondrial membrane potential (MMP), plasma membrane acrosome integrity (PMAI), and viability were evaluated by flow cytometry (Beckman Coulter, United States). DNA fragmentation was evaluated by the alkaline single-cell gel electrophoresis method (COMET assay). Biochemical analyses were carried out by spectrophotometric methods.

### 2.2 Motility and kinetic characteristics

The analysis was performed using a CASA system (Nikon Eclipse 50i; Japan) and a heating plate. The curvilinear velocity (VCL) parameter was classified five categories: static (<10 μm/s), slow (10–45 μm/s), medium (45–75 μm/s), fast (>75 μm/s), and progressive (>75% straightness). Before analysis the samples were thawed, an amount of 5 μL was then put on a slide, covered with a cover slide, and heated on the microscope’s heating plate to 37°C. At least 200 spermatozoa were counted and examined in five different microscopic zones for each sample (23). Frozen–thawed semen samples were analyzed in terms of total motility (TMOT, %), progressive motility (PMOT, %), straightness (STR, %), curvilinear velocity (VCL, μm/s), linearity (LIN, %), straight-line velocity (VSL, μm/s), average path velocity (VAP, μm/s), wobble (WOB, %), beat-cross frequency (BCF, Hz), and amplitude of lateral head displacement (ALH, μm/s).

### 2.3 Flow cytometric analyses

Tests were performed using a CytoFLEX flow cytometer (Beckman Coulter, CA, United States) equipped with emission filters at 610 ± 20 nm, 585 ± 42 nm, and 525 ± 40 nm, as well as a 50 mW (488 nm) laser output. An average of 10,000 spermatozoa were analyzed for each test. Pseudo-color plots were used to compare the side scatter area (SSC-A) to the forward scatter area (FSC-A) of the sperm cells to facilitate the selection process. Forward scatter height (FSC-H) and forward scatter area (FSC-A) were used to exclude doublets from the analysis (24). All aliquots of 50 μL were made from the stain stock solutions prepared using DMSO and kept at −20°C.

A FITC/PNA-PI staining protocol was used to detect spermatozoon parameters using the method described by İnanç et al. (25), 100 μg/mL of FITC-PNA (Sigma, L7381), and 2.99 mM propidium iodide (PI, L7011 Molecular Probes, Invitrogen). The sperm cell concentration was adjusted to 5×10^6^ by diluting the semen samples with 492 μL of PBS. Then, 5 μL of FITC and 3 μL of PI were added to the mixture, and the mixture was incubated at 37°C for 15 min in a dark environment. After the analysis, the FITC- and PI-populations were recorded as PMAI (%). The MMP of the sperm was determined using 5,5′,6,6′ tetrachloro-1,1′,3,3′-tetramethyl benzimidazolyl-carbocyanine iodide (JC-1) (25). The concentration of JC-1 (T3198 molecular probes, Invitrogen) was 0.153 mM. The sperm cell concentration was adjusted to 5×10^6^ by diluting the semen samples with 495 μL of PBS. 5 μL of JC-1 was added to the mixture, which was then incubated at 37°C for 15 min in the dark. Spermatozoa were evaluated based on their HMMP status after the analysis. The viability of sperm cells was determined using the SYBR and PI protocols (5). We used 1:10 SYBR and 2.99 mM PI from among Invitrogen’s L7011 Molecular Probes. The sperm cell concentration was adjusted to 5×10^6^ by diluting the semen samples with 492 μL of PBS. 5 μL of SYBR-14 and 3 μL of PI were introduced into the mixture, which was then subjected to incubation at 37°C for a duration of 15 min in the dark. After the analysis, the SYBR+ and PI-populations were recorded as viable (plasma membrane integrity, %).

### 2.4 Evaluations of chromatin fragmentation

The method reported by Gündoğan et al. (26) was used to conduct a COMET assay to test sperm chromatin integrity. The samples that were stained and processed were examined using a microscope (Olympus CX31) equipped with a fluorescence attachment. The Comet Score software (TriTek, V. 1.5) was utilized to evaluate the sperm cells. A total of 200 sperm cells were examined and evaluated across six distinct microscopic zones.

### 2.5 Biochemical analyses

Malondialdehyde (MDA) was used in to quantify the degree of lipid peroxidation (LPO), in accordance with the procedure described by Draper and Hadley (27). The MDA concentration was assessed based on the reaction between lipid peroxides and thiobarbituric acid, followed by the measurement of absorbance at 532 nm. The concentration of MDA was determined in units of nanomoles per milliliter (nmol/mL). Ellman’s method was employed to determine the quantity of reduced glutathione (GSH), and the resulting concentration was computed as mg/dL with the methodology reported by Hissin and Hilf (28). Total antioxidant status (TAS) measurements were made using a colorimetric test kit (REL Assay Diagnostics, Gaziantep, TR). The experiment entailed a reduction of the oxidized radical 2,2-azino-bis-3-ethylbenzothiazoline-6-sulfonic acid (ABTS) through the action of the antioxidant compounds present in the samples, leading to visible alterations in color. A spectrophotometer was utilized to measure color intensity at a wavelength of 660 nm, and the outcomes are expressed in units of mmol/L. Total oxidant status (TOS) measurements were made using a colorimetric test kit (REL Assay Diagnostics, Gaziantep, TR). The methodology employed in this study involved the assessment of the oxidation of Fe^+2^ to Fe^+3^ by the usage of oxidizing agents. The concentration of the solution was determined in μmol/L and measured via the spectrophotometric analysis method at a wavelength of 660 nm. Finally, the oxidative stress index (OSI) was calculated using the formula OSI = [(TOS)/ (TAS × 100)] (29).

### 2.6 Statistical analyses

Prior to conducting the significance tests, the data were assessed for normality using the Shapiro-Wilks test. Levene’s test was used to determine the homogeneity of the variances. ANOVA was used to statistically test the differences between groups of data. The Duncan test was applied to evaluate differences between groups. Descriptive statistics are presented as “mean ± standard error of the mean” (Mean ± SE). Using the SPSS 13.0 package program, the results of all statistical analyses were evaluated with a maximum error margin of 5%. A *p*-value of 0.05 was accepted as the threshold of statistical significance.

## 3 Results

The progressive motility rate showed statistically significant differences among the groups (*p* < 0.05). In comparison to C, B1 and B2 had contrasting effects on both total and progressive motility (Table 1; *p* < 0.05). B0.5 achieved numerically better results compared to C in terms of VSL and hyperactivity. The results for VAP, VSL, VCL, BCF, and WOB in B2 were found to be significantly lower compared to those in C and B0.5 (*p* < 0.05). It was determined that the higher doses of treatment (1 and 2 mM) did not have positive effects on kinetic parameters. B0.5 produced significantly better HMMP and viability outcomes than the other B groups (*p* < 0.05), but C had the highest HMMP results (*p* < 0.05). The highest PMAI value was found in B1 (*p* < 0.05). The results on the PMAI, HMMP, and viability parameters showed that the highest concentration of B (2 mM) did not prevent damage to the ram semen caused by the cryopreservation process (*p* > 0.05; Table 2). B0.5 and B1 had significantly lower levels of chromatin damage compared to C (*p* < 0.05). Except for B2, chromatin integrity was preserved in all B groups compared to C (*p* < 0.05; Table 3). As seen in Table 4, there were no significant changes in GSH, MDA, TOS, and OSI levels among the groups (*p* > 0.05). While the highest GSH activity and OSI and the lowest MDA and TAS levels were in B2 among all groups, (*p* < 0.05).

**Table 1:** Sperm motility and kinetic parameters of frozen–thawed ram semen.

Parameters	C	B0.5	B1	B2	*p*
Progressive motility (%)	12.47 ± 1.48^b^	17.50 ± 1.99^a^	8.21 ± 1.01^c^	2.85 ± 0.46^d^	*
Total motility (%)	59.52 ± 2.87^a^	66.80 ± 1.96^a^	38.48 ± 4.27^b^	16.16 ± 1.31^c^	*
VAP (μm/s)	35.94 ± 1.43^a^	35.38 ± 2.66^a^	33.05 ± 1.50^a^	27.19 ± 2.20^b^	*
VSL (μm/s)	21.83 ± 1.01^a^	21.92 ± 1.80^a^	21.23 ± 1.27^a^	16.65 ± 1.85^b^	*
VCL (μm/s)	57.26 ± 1.66^a^	56.50 ± 3.14^a^	54.52 ± 1.95^ab^	48.35 ± 2.00^b^	*
ALH (μm/s)	2.38 ± 0.07	2.43 ± 0.09	2.40 ± 0.11	2.36 ± 0.07	–
BCF (Hz)	8.42 ± 0.35^a^	7.64 ± 0.33^ab^	6.81 ± 0.31^b^	5.26 ± 0.51^c^	*
LIN (%)	38.19 ± 1.21	37.52 ± 1.78	38.64 ± 2.52	32.83 ± 2.37	–
STR (%)	58.76 ± 0.95^ab^	58.38 ± 0.79^ab^	59.87 ± 2.22^a^	53.96 ± 2.26^b^	*
WOB (μm/s)	61.72 ± 1.12^a^	60.97 ± 2.24^a^	59.05 ± 1.91^ab^	54.12 ± 2.23^b^	*
Hyperactivity	0.98 ± 0.21^ab^	1.19 ± 0.28^ab^	1.71 ± 0.37^a^	0.53 ± 0.19^b^	*

^a,b,c,d^ Different superscripts on the same row demonstrate significant differences (*p < 0.05). No significant difference (*p* > 0.05). Straight line velocity (VSL), curvilinear velocity (VCL), straight line linearity (LIN) [(VSL/VCL) × 100], average path velocity (VAP), straightness (STR) [(VSL/VAP) × 100], amplitude of lateral head displacement (ALH), cross beat frequency (BCF), wobble (WOB) [(VAP/VCL) × 100]. Mean (±SE).

**Table 2:** PMAI, HMMP and SYBR14+ activities in frozen–thawed ram semen.

Parameters	C	B0.5	B1	B2	*p*
PMAI (%)	17.87 ± 2.39^b^	20.89 ± 1.19^b^	30.00 ± 2.14^a^	11.31 ± 0.80^c^	*
HMMP (%)	15.51 ± 0.42^a^	13.26 ± 0.47^b^	11.66 ± 0.49^c^	5.69 ± 0.29^d^	*
Viability (%)	68.79 ± 1.09^ab^	72.46 ± 1.03^a^	67.76 ± 1.29^b^	59.66 ± 2.00^c^	*

^a,b,c,d^ Different superscripts on the same row demonstrate significant differences (*p < 0.05). High mitochondrial membrane potential (HMMP), plasma membrane and acrosome integrity (PMAI), viability (plasma membrane integrity). Mean (±SE).

**Table 3:** DNA damage values in frozen–thawed ram semen.

Parameters	C	B0.5	B1	B2	*p*
Tail length (μm/s)	25.23 ± 0.92^a^	14.00 ± 0.99^c^	14.07 ± 0.83^c^	18.46 ± 1.29^b^	*
Tail DNA (%)	36.67 ± 3.13^a^	23.75 ± 2.17^b^	22.72 ± 2.40^b^	34.70 ± 0.96^a^	*
Tail moment (μm/s)	20.66 ± 1.86^a^	15.51 ± 1.78^bc^	12.36 ± 1.05^c^	18.91 ± 0.75^ab^	*

^a,b,c,^ Different superscripts on the same row demonstrate significant differences (*p < 0.05). Mean (±SE).

**Table 4:** GSH and MDA activities, TAS, TOS, and OSI values in frozen–thawed ram semen.

Parameters	C	B0.5	B1	B2	*p*
GSH (mg/dL)	45.30 ± 2.64	40.51 ± 1.26	40.42 ± 0.87	46.15 ± 2.49	–
MDA (nmol/mL)	5.90 ± 0.08	5.96 ± 0.05	5.81 ± 0.15	4.15 ± 0.06	–
TAS (mmol/L)	3.42 ± 0.14^ab^	3.53 ± 0.11^ab^	3.55 ± 0.02^a^	3.22 ± 0.07^b^	*
TOS (μmol/L)	8.33 ± 0.48	7.84 ± 0.27	7.94 ± 0.20	8.02 ± 0.42	–
OSI (TOS/TASx100)	24.37 ± 1.15	22.31 ± 1.02	22.37 ± 0.55	24.81 ± 0.89	–

^a,b^ Different superscripts on the same row demonstrate significant differences (*p < 0.05).^-.^ No significant difference (*p* > 0.05). Malondialdehyde (MDA), glutathione (GSH), total antioxidant status (TAS), and total oxidant status (TOS), oxidative stress index (OSI). Mean (±SE).

## 4 Discussion

Successful cryopreservation of sperm cells requires an understanding of the changes that occur throughout the process, and there have been several studies on this topic (30–32). Research efforts have concentrated on diverse attributes, including but not limited to the motility and viability of sperm, the integrity of sperm cell membranes, and chromatin integrity. One of the primary challenges in establishing uniform protocols for the cryopreservation of sperm is inter-individual variability in the response of sperm cells to identical freezing procedures. Studies in the relevant field aim to gain an insight into the anticipated reaction of sperm cells to cryopreservation. Secondly, they strive to create customized cryopreservation protocols tailored to optimize the effectiveness of their technique. The primary goal is to enhance spermatological characteristics during freeze–thaw cycles (31). Previous studies have attempted to explain the processes that regulate B. These are the blockage of the Fas/FasL pathway (reducing cell apoptosis), the activation of the nuclear factor erythroid 2-related factor 2 (Nrf2) (promoting anti-oxidative stress effects), the inhibition of the Nrf2/HO-1 (heme oxygenase 1) signaling pathway, and the regulation of the mTOR pathway (cell regulator) (33–36). This study aimed to determine whether the addition of B at three different doses improves the resistance of sperm cells to cryodamage during cryopreservation. It was found that the 0.5 mM dose of B (B0.5) prevented damage to progressive motility, in agreement with increased doses of B also caused toxicity (35). This might be due to the use of low doses of B compared to high doses shows its effect by blocking the Fas/FasL pathway. This result confirmed the results of previous studies, indicating that B shows dose-dependent effects, as demonstrated by Fan et al. (37). B has not been used in sperm extenders, and thus, we are unable to discuss these results along with the results of similar studies. On the other hand, the data in our study were in accordance with prior information that antioxidant agents that are added to semen extenders could promote progressive motility in a dose-dependent manner (5, 38, 39). B has different effects on cell-induced apoptosis, whereas it inhibits metastasis, on the other hand, and protects the viability of mitochondria and cells (40–42).

Comparable outcomes were obtained in B1 group with regard to plasma membrane integrity. This could have been caused by B’s effect in the activation of the signaling pathway Nrf2 and regulation of the mTOR pathway. Sperm cells, unlike other cells, have a unique structure in terms of their sensitivity to damage by LPO and defense reactions (43). According to Kovalski et al. (44), sperm cells cannot repair themselves following damage resulting out of LPO. The absence of cytoplasmic enzymes within these cells creates an imbalance favoring the production of ROS, leading to the heightened susceptibility of semen to oxidative stress. In our study, while B0.5 positive outcomes in terms of progressive motility, it did not have positive results regarding oxidative stress parameters. Although B did not have a positive effect on oxidative stress parameters, B0.5 had a positive effect on motility, suggesting that B may provide an intrinsic antioxidant property by donating electrons to the spermatozoon plasma membrane. Among other studies in which contradictory results have been presented, a correlation was observed between elevated levels of ROS and reduced motility (45). Alahmar (46) argued that the correlation between ROS and reduced motility can be attributed to a sequence of events that culminate in a decline in the phosphorylation of axonemal proteins and the subsequent immobilization of sperm. The primary function of antioxidants in the human body is to mitigate the impact of oxidative processes (47). This can be achieved either through direct means, such as the catalysis of enzymatic reactions, or indirectly, by acting as compounds that neutralize free radicals and interrupt chain reactions. B could effectively inhibit the generation of ROS by reducing hydrogen peroxide (37). Moreover, similar studies have added different antioxidants to sperm extenders before freezing (5, 38, 48–50). B could also reduce the expression of caspase 9 and caspase 3 (41). Beside these effects, B promotes the viability of neuroblastoma cells and prevents the apoptosis-inducing effects of hydrogen peroxide on neurocytes. This effect is probably related to the upregulation of deacetylase sirtuin 1 (SIRT1), which is responsible for cellular functions that are favorable for the lifespan of organisms and is often considered an “anti-aging” enzyme, and the downregulation of caspase 3 (12, 41). In this study, B0.5 did not show significant improvements in viability. This result contradicted the results found by Pan et al. (41) and Wang et al. (15). Oxidative damage to mitochondrial DNA is a well-documented phenomenon occurring in aerobic cells, including sperm cells, due to their abundance of mitochondria. Prior research has indicated that the application of B at minimal concentrations can effectively lower the rate of apoptosis in bovine mammary epithelial cells (51). These results were in accordance with our results. The B doses in B0.5 and B1 protected the sperm from chromatin damage, while the dose in B2 did not. It is thought that B could block H_2_O_2_-induced DNA damage by inhibition of DNA tail formation and γH2AX phosphorylation. Thereby protect it from oxidative damage. Fan et al. (37) suggested that an excessive B dose beyond the physiological concentration may induce cellular stress, leading to cellular toxicity. Similar findings have been reported in the flow cytometric assessments of plasma membrane acrosome integrity, viability, and mitochondrial membrane status.

## 5 Conclusion

The results of this study suggested that the 0.5 mM dosage of B could preserve the progressive motility and chromatin integrity of ram sperm cells after the freeze-thawing process. Additional comprehensive studies on the function of B in the progression of cryoinjury and its underlying mechanisms are expected to yield novel insights and approaches for the improvement of the semen cryopreservation process.

## Data availability statement

The original contributions presented in the study are included in the article/supplementary material, further inquiries can be directed to the corresponding author/s.

## Ethics statement

The animal studies were approved by the Animal Research Ethics Committee of Afyon Kocatepe University (Approval No. 49533702/333). The studies were conducted in accordance with the local legislation and institutional requirements. Written informed consent was obtained from the owners for the participation of their animals in this study.

## Author contributions

FA: Investigation, Resources, Writing – original draft. ŞG: Data curation, Investigation, Resources, Writing – original draft. MG: Investigation, Resources, Writing – original draft. Mİ: Investigation, Resources, Writing – original draft. KO: Investigation, Writing – original draft. BD: Investigation, Writing – original draft. DY: Investigation, Resources, Writing – original draft. UT: Investigation, Methodology, Project administration, Resources, Writing – review & editing.
